# Intraoperative PTH cutoff definition to predict successful parathyroidectomy in secondary and tertiary hyperparathyroidism

**DOI:** 10.5935/1808-8694.20130088

**Published:** 2015-10-08

**Authors:** Monique Nakayama Ohe, Rodrigo Oliveira Santos, Ilda Sizue Kunii, Aluizio Barbosa Carvalho, Márcio Abrahão, Murilo Catafesta das Neves, Marise Lazaretti-Castro, Onivaldo Cervantes, Jose Gilberto Henriques Vieira

**Affiliations:** a MSc. PhD student in Endocrinology and Metabology - Paulista School of Medicine - Federal University of São Paulo (EPM-UNIFESP) (Graduate Student in Endocrinology and Metabology - EPM-UNIFESP).; b Phd (Professor at the Department of ENT-HNS at EPM-UNIFESP. Deputy Chief - Department of ENT-HNS - EPM-UNIFESP).; c MSc. Biomedic - Laboratory of Endocrinology and Metabology - EPM-UNIFESP.; d PhD in Nephrology (Associate Professor of Nephrology).; e Senior Associate Professor - Department of ENT-HNS - EPM-UNIFESP (Head of the HNS Program - EPM-UNIFESP).; f Head and Neck Surgeon (Assistant at the HNS Program - EPM-UNIFESP).; g Senior Associate Professor of Endocrinology and Metabology - EPM-UNIFESP (PhD; Professor of Endocrinology and Metabology - EPM-UNIFESP. Head of the Osteometabolic Ward Disorders Course in the Endocrinology and Metabology Program - EPM-UNIFESP).; h Senior Associate Professor - Department of Otorhinolaryngology and Head and Neck Surgery.; i PhD (Professor of Endocrinology and Metabology - EPM-UNIFESP).

**Keywords:** hyperparathyroidism, hyperparathyroidism, secondary, parathyroid hormone, parathyroidectomy

## Abstract

In order to improve success rates in surgery of renal hyperparathyroidism, we evaluated intraoperative PTH (IOPTH) measurement utility.

**Method:**

86 patients underwent total parathyroidectomy with intramuscular presternal autotransplantation from 04/2000 to 10/2009 and were followed for 26.5 months on average (prospective cohort). Patients were divided in secondary (SHPT) and tertiary hyperparathyroidism (THPT). SHPT group was composed by patients under dialysis treatment, THPT group included renal grafted ones. IOPTH (Elecsys-PTH-Immunoassay/Roche) was measured at anesthesia induction (IOPTH-0’) and 20 minutes (IOPTH-20’) after parathyroidectomy.

**Results:**

80.2% (69/86) presented with 80% decrease or more in the IOPTH-20’ and all were cured. In 11/86 patients (12.7%), a lower IOPTH-20’ drop (70-79%) was observed, and 2 of them (18.1%) failed to cure. 6/86 (6.9%) patients presented IO-PTH-20’ decrease of less than 70%: two were cured, in three a supernumerary/ectopic parathyroid was found and removed, and in one of these six patients, surgery was finished after 4-gland excision and the patient failure to cure.

**Conclusion:**

IOPTH-20’ decrease of 80% or more compared to IOPTH-0’ predicts cure in all renal patients throughout follow-up. A decay of less than 70% points to missed or hyperfunctioning supernumerary gland and is predictive of surgical failure in 66.6%. A marginal IOPTH drop of 70-79% leaves the decision whether or not surgery should be continued up to the experienced surgeon.

## INTRODUCTION

Secondary and tertiary hyperparathyroidism results from parathyroid gland hyperplasia and excess parathyroid hormone (PTH) secretion^1^. Despite recent advances in medical therapy, parathyroid surgical excision can be necessary in a considerable number of renal patients^2^. The best surgical approach for renal hyperparathyroidism is still debated, and controversy remains raising relevant questions regarding treatment election, since post-surgical recurrence or the risk of definitive hypoparathyroidism are to be avoided. Total parathyroidectomy with parathyroid tissue autotransplantation is a well-accepted technique for the management of these patients^3^, ^4^, ^5^, ^6^. The surgical procedure involves bilateral neck exploration, seeking for total removal of abnormal parathyroid tissue. Persistence and/or recurrence indicate an inadequate resection of hyperfunctioning tissue. Reoperation is reported to be necessary for recurrent renal hyperparathyroidism in about 15% of cases, mostly due to the presence of supernumerary glands, inadequate initial parathyroidectomy, or the continued hyperplasia of remnant tissue^7^, ^8^.

Since its introduction, intraoperative PTH monitoring (IO-PTH) has been performed in patients with primary hyperparathyroidism in several medical centers, becoming a well-recognized predictor of surgical success in in these patients^9^, ^10^, ^11^, ^12^, ^13^. By resorting to IO-PTH measurements, the surgeon can confirm total removal of the abnormal parathyroid glands, avoiding overlooking remaining or supernumerary hyperfunctioning glands. However, differently from patients with primary hyperparathyroidism, the role of IO-PTH monitoring in the surgical treatment of renal hyperparathyroidism is less established^14^. There is no standard definition of IO-PTH decay in renal hyperparathyroidism surgical treatment, since impaired renal function and delayed renal clearance of PTH can interfere in IO-PTH monitoring. Besides, few large series using IO-PTH monitoring in renal patients have been published^14^.

The aim of the present study was to evaluate the utility of intraoperative PTH monitoring in dialysis or kidney transplanted patients with secondary and tertiary hyperparathyroidism. Our aim was to define an IO-PTH cutoff value in order to avoid missed and/or supernumerary parathyroid glands, improving surgical success rates.

## METHOD

### Study design

This is a prospective study on a cohort of patients operated at a university referral center. All surgeries were performed by the same surgeon. This investigation was approved by Ethics Committee of our institution (approval No. CEP 886/00) and patients gave their informed consent prior to their inclusion in the study.

### Patients

Eighty-six renal patients underwent total parathyroidectomy with intramuscular presternal autotransplantation^6^ from April 2000 to October 2009, São Paulo, Brazil. Patients were followed in our institution and were referred to surgical treatment in face of: persistent hypercalcemia not responsive to medical interventions and/or persistent hyperphosphatemia despite the continued use of dietary phosphorus restriction and phosphate-binding agents, symptoms as intractable pruritus, severe bone pain, fractures or high risk of fracture, skeletal deformities, extra skeletal calcifications, development of calciphylaxis and radiographic evidences of renal osteodystrophy.

They were divided in secondary and tertiary hyperparathyroidism groups. Secondary hyperparathyroidism is characterized as an acquired disorder seen in end-stage renal disease, in which the uremic state presents a continuous stimulus to the parathyroid glands. We included in the SHPT group, patients under dialysis treatment who presented severe hyperparathyroidism with normal or high serum calcium levels. Tertiary hyperparathyroidism group was composed by renal patients with kidney transplant with nonsuppressible parathyroid hyperplasia, with persistent increased intact PTH levels and hypercalcemia even after restoration of normal renal function. Hypercalcemia after kidney transplantation is usually due to hyperparathyroidism that persists from the preceding chronic kidney disease period^15^.

Serum ionized calcium (iCa), phosphorus (P), alkaline phosphatase (AP), and intact parathyroid hormone (iPTH) were measured before parathyroidectomy (Table 1) and yearly after surgery in all patients from both groups.

**Table 1:** Patient demographic characteristics.

	SHPT	THPT
N	52	34
Age (years)	42.9 (14-64)	43.5 (24-63)
Female/male	29/23	19/15
Years in dialysis	7.4 (0.75-15)	6 (0.5-13)
Years of renal graft	-	2.8 (0.1-5.3)
Follow-up (months)	27.8 (6-65)	24.5 (6-67)

Data on average and range (parenthesis).

Surgical cure was defined as restoration of serum calcium and iPTH levels as expected for each renal function stage, throughout the first 6 months after surgery^15^. Recurrence after surgery was defined when high levels of iPTH were observed throughout late post-operative follow-up (1 year after surgical procedure) that failed to respond to medical/pharmacological management. Definitive hypoparathyroidism was described when PTH measurements under 10 pg/mL endures 1 year after parathyroidectomy, with normal or low serum calcium levels under vitamin D and oral calcium replacement.

### Methods

Total parathyroidectomy with intramuscular presternal autotransplantation^6^ was performed in 86 patients from April 2000 to October 2009. Patients were evaluated throughout a 26.5-month follow up on average (range 6-67 months).

Intra-operative PTH (IO-PTH) was performed in attempt to confirm total removal of the parathyroid glands, so as to avoid overlooking remaining or supernumerary hyperfunctioning glands. IO-PTH was measured by resorting to the Elecsys PTH Immunoassay (Elecsys 1010 System, Roche, Mannheim, Germany). The time required to carry out the assay is 9 minutes and reference values are 10-70 pg/mL. Peripheral venous blood sample (4.0 mL) was obtained immediately after induction of anesthesia and 20 minutes after removal of all parathyroid glands^12^, ^13^.

Phosphorus, total alkaline phosphatase and creatinine were measured by means of standard automatic assays (Hitachi 912, Roche). Serum ionized calcium was measured by using an ion-specific electrode (AVL 9180 Electrolyte Analyzer, Roswell, Georgia, USA). Parathyroid hormone was measured by immunometric assay (Immulite, DPC, MedLab).

## RESULTS

Overall, 86 patients underwent total parathyroidectomy with presternal intramuscular autotransplantation with IO-PTH monitoring. Patients were evaluated in a prospective way in an average follow-up of 26.5 months (ranging from 6 to 67 months). There were 52 patients in the SHPT group and 34 in THPT group. Patients from SHPT group presented an average of 7.4 years under dialysis treatment before parathyroidectomy, and THPT group comprised patients who had undergone dialysis treatment for an average of 6 years before receiving a kidney graft, with an average of 2.8 years of functional renal graft before parathyroidectomy. Patient demographic characteristics are depicted in Table 1 and patient pre-operative biochemical data are depicted in Table 2.

**Table 2:** Patient pre-operative laboratory data.

	SHPT (N = 52)	THPT (N = 34)
iCa	1.38 mmol/L (1.19-1.83)	1.59 mmol/L (1.42-1.75)
iPTH	1682.1 pg/mL (516-2500)	440.9 pg/mL (109-1758)
P	6.5 mg/dL (4.1-11.8)	2.8 mg/dL (1.9-6.3)
AP	603.2 U/L (150-1639)	289.9 U/L (66-1304)
Cr	-	1.7 mg/dL (1-9.2)

SHPT: Secondary hyperparathyroidism; THPT: Tertiary hyperparathyroidism, iCa: Serum ionized calcium, P: Phosphorus; AP: Alkaline phosphatase; iPTH: Intact parathyroid hormone. Reference values: iCa: 1.11-1.40 mmol/L; P:2.3-4.6 mg/dL; AP: 35-129 U/L; iPTH: 10-65 pg/mL. Data on average and range (parenthesis).

Regarding surgical findings, at least four parathyroid glands were identified and excised in all 86 patients, and supernumerary glands were found in three (the 5^th^ parathyroid was located in the cervical region in two patients and in the thymus in one).

Patients under dialysis treatment, who were cured after surgery, presented IO-PTH basal levels of 1591.5 pg/mL on average, and IO-PTH 20 minutes after total parathyroidectomy of 208.9 pg/mL on average, with 86% decrease. Among renal-grafted cured patients, basal IO-PTH was 540 pg/mL on average, and 65 pg/mL on average, 20 minutes after total parathyroidectomy, with a 87.3% decrease on average (Table 3).

**Table 3:** Intra-operative PTH decay in cured patients.

	SHPT	THPT
IO-PTH	1591.5 pg/mL (318-4659)	540 pg/mL (120-2515)
IO-PTH 20’	208.9 pg/mL (29-823)	65 pg/mL (13-329)
% decrease	86% (67.8-93.5)	87.3% (72.6-96.4)
Post-op iCa	1.22 mmol/L (0.97-1.38)	1.20 mmol/L (1.04-1.35)
Post-op iPTH	76.4 pg/mL (5-497)	60.8 pg/mL (17-142)
Follow up (months)	27.8 (6-67)	25 (6-67)

IO-PTH 0’: Intra-operative PTH in anesthesia induction; IO-PTH 20’: Intra-operative PTH 20 minutes after total parathyroidectomy; SHPT: Secondary hyperparathyroidism; THPT: Tertiary hyperparathyroidism iCa: Serum ionized calcium, iPTH: Intact parathyroid hormone; Post-op iCa and iPTH: Post-operative measurements of iCa and PTH. Data on average and range (parenthesis).

**Graph 1:**
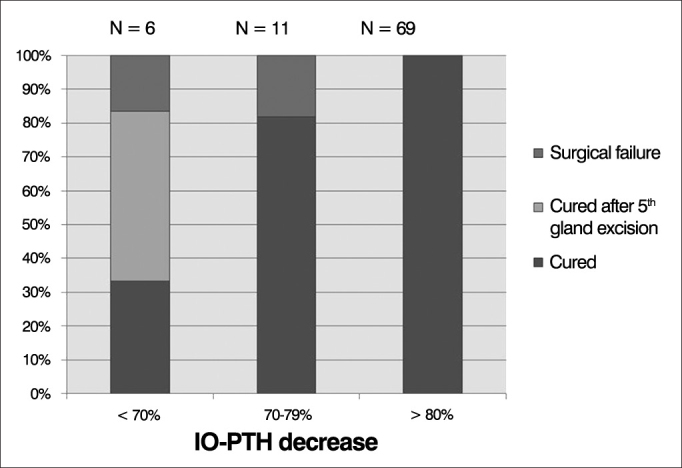
IO-PTH percent decrease and surgical outcome.

Overall, 69 patients (80.2%) presented 80% decrease or more in IO-PTH levels, and all patients were cured (Graph 1). Eleven patients (12.7%) presented a drop of 70-79%, and among them, two patients failed to cure (IO-PTH decrease of 76.9% and 77.2%) (Table 4, Graph 1). IO-PTH prevented surgical failure in three patients, in whom a 58.5%, 51.8% and 62% IO-PTH drop was considered insufficient. Surgical exploration was continued and a supernumerary cervical parathyroid gland was removed in two and supernumerary ectopic parathyroid in the thymus was resected in one (Table 5, Graph 1). One patient presented IO-PTH drop of 67.4% (Table 5, patient 4). This drop was considered sufficient and surgery was finished after excision of four parathyroid glands. Post-operative PTH levels remained high in the early post-operative follow-up, and patient was considered not cured (Table 5, patient 4).

**Table 4:** Patients with 70-79% IO-PTH decrease who failure to cure.

	IO-PTH 0’	IO-PTH 20’	Decrease	6 months post-op iCa PTH	20 months post-op iCa PTH
Patient 1	2481 pg/mL	573 pg/mL	76.9%	1.34 mmol/L 429 pg/mL	1.22 mmol/L 810 pg/mL
Patient 2	553 pg/mL	126 pg/mL	77.2%	1.60 mmol/L 179 pg/mL	-

Reference values: iCa: 1.11-1.40 mmol/L; iPTH: 10-65 pg/mL.

**Table 5:** Patients with IO-PTH decrease less than 70%.

Pat	IO-PTH 0’	IO-PTH 20’	%	Surgical findings	6 mths post-op iCa PTH	20 mths post-op iCa PTH
1	1913 pg/mL	782 pg/mL	58.5%	5^th^ PTX	-	-
2	1295 pg/mL	624 pg/mL	51.8%	5^th^ PTX (thymus)	-	-
3	327 pg/mL	124 pg/mL	62%	5^th^ PTX	-	-
4	860 pg/mL	280 pg/mL	67.4%	Surgery finished after excision of 4 glands	1.26 605 pg/mL	1.24 1320 pg/mL

Pat: Patient; %: Percent decrease; mths: Months; 5^th^ PTX: Supernumerary parathyroid. Reference values: iCa: 1.11-1.40 mmol/L; iPTH: 10-65 pg/mL.

Definitive hypoparathyroidism was observed in two patients, one in each group (2.3% among overall patients).

## DISCUSSION

The rapid intraoperative determination of intact parathyroid hormone has become widely established in primary hyperparathyroidism surgical treatment, in which the removal of all hyperfunctioning parathyroid tissue is confirmed by a prompt decline in circulating iPTH within 5 to 10 minutes^4^, ^12^, ^13^, ^16^, ^17^, ^18^, ^19^. The intraoperative decay of circulating iPTH in renal patients is reproducible but markedly slower than in the patients with normal renal function. This is in accordance with the increased half-life of iPTH in renal failure (6.6 minutes versus 2.2 minutes)^20^. Studies with rapid intraoperative PTH measurement in secondary hyperparathyroidism demonstrated a stepwise decrease in circulating iPTH corresponding to sequential gland resection^12^, ^21^; and those findings together with a higher incidence of supernumerary/ectopic parathyroid glands in renal hyperparathyroidism^7^, ^8^, point to IO-PTH measurement as an interesting tool in an attempt to avoid surgical failure.

Differences between SHPT and THPT patients might be pointed out regarding to IO-PTH decay profile: patients under dialysis treatment may present a slower IO-PTH decay compared to kidney-grafted ones, in which renal function is restored. Moreover, THPT patients resemble primary hyperparathyroidism patients, with hypercalcemia and normal renal function. However, THPT is a multiglandular disease (in opposition to uniglandular primary hyperparathyroidism typical disease), requiring different surgical approach, probably interfering to IO-PTH kinect analysis. That coupled with the difficulty to assume completely normal renal function years after kidney-graft transplantation, turns THPT more similar to SHPT than to primary hyperparathyroidism. Due to the increased iPTH half-life in renal failure patients, a 20-minutes IO-PTH measurement was chosen, a delayed measurement in comparison to the 10-minutes IO-PTH measurement established in primary hyperparathyroidism. Thereafter, despite of potential differences among SHPT and THPT patients, data were studied coupling both groups.

A few studies with small series and/or limited follow-up evaluating IO-PTH percentile decrease in renal population have been published^8^, ^13^, ^14^, ^22^. Some authors have observed IO-PTH decay of 50 to 60% at 10 to 20 minutes after parathyroidectomy as a reliable predictor of postoperative cure^23^, ^24^, ^25^. Other authors expected more than 80% decay in IO-PTH measurements 15 minutes after parathyroidectomy in order to assure surgical cure^26^. Our previous experience showed that a decrease of 70% 20 minutes after parathyroidectomy indicates a successful surgical procedure^13^.

In this study, IO-PTH decay of 80% or more 20 minutes after parathyroidectomy assured surgical cure in all patients. Overall, 69 (80.2%) patients presented IO-PTH decay of 80% or more, and all were cured. Six patients (6.9%) presented less than 70% decay in IO-PTH, and among them, four (66.6%) presented a supernumerary/ectopic parathyroid removed (three of them) or remained not cured (one patient).

Taking into account these results, we conclude that 80% decay or more in IO-PTH measurement 20 minutes after total removal of hypersecretory parathyroid tissue is expected and predicts 100% of cure. Moreover, IO-PTH decay under 70% is associated with 66.6% of surgical failure, most of which associated with a supernumerary/ectopic parathyroid gland.

Special concerns have to be raised about the “marginal” IO-PTH drop - between 70% to 79% - observed in 11 patients (12.7%), since two patients among them (18.1%) failed to cure. Particular questions remain unsolved: how can the surgeon recognize these patients? To what extent should surgery continue in the search for further hypersecretory glands? Those questions remain unanswered, and it is up to the experienced surgeon to point out when and how to conclude the surgical procedure, based on patient clinical aspects and ongoing surgical findings.

Limitations of this study are related to the heterogeneous characteristics of renal patients with their particular clinical and laboratory findings, and considering them as a group presents a real challenge. Another limitation relates to the severity of our patients: the marked illness and long-lasting renal condition make them a special group whose outcome would not be expected in ordinary patients with renal chronic disease. In addition, levels of 25OH vitamin D were not available.

## CONCLUSION

A decrease of IO-PTH of 80% or more from preoperative baseline predicts cure in all renal patients throughout follow-up. IO-PTH decay under 70% is in agreement with a missed or hyperfunctioning supernumerary gland and is predictive of surgical failure in 66.6%. The marginal IO-PTH drop of 70-79% leads the decision whether or not the surgery should be continued up to the experienced surgeon.
